# Editorial: Innate and adaptive immunity in normal and adverse pregnancy

**DOI:** 10.3389/fimmu.2026.1812570

**Published:** 2026-03-20

**Authors:** Hadida Yasmin, Chiara Agostinis, Roberta Bulla, Uday Kishore

**Affiliations:** 1 Department of Zoology, Cooch Behar Panchanan Barma University, West Bengal, Cooch Behar, India; 2 Institute for Maternal and Child Health, IRCCS Burlo Garofolo, Trieste, Italy; 3 Department of Life Sciences, University of Trieste, Trieste, Italy; 4 Department of Veterinary Medicine, United Arab Emirates University, Al Ain, United Arab Emirates; 5 Zayed Centre for Health Sciences, United Arab Emirates University, Al Ain, United Arab Emirates

**Keywords:** adverse pregnancy, complement, maternal-fetal interface, NK cells, T cells

## Abstract

**Figure:**
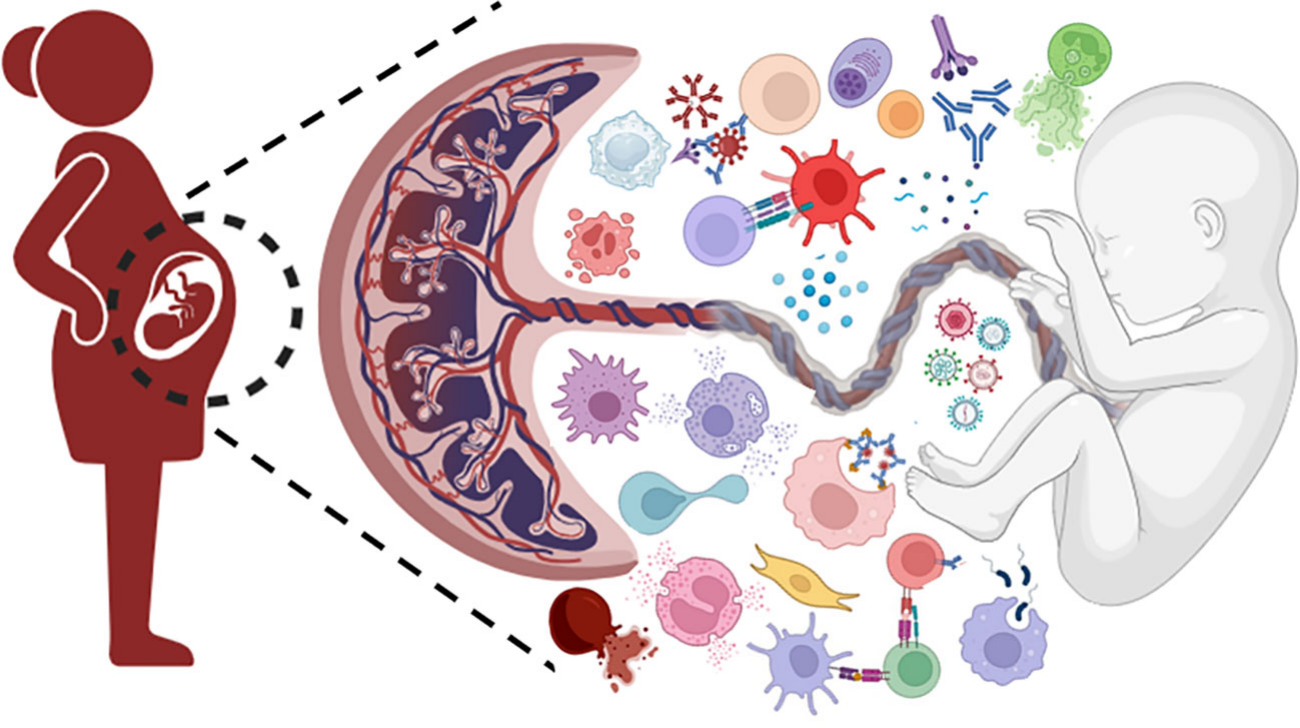

During pregnancy, the maternal immune system must engage in a fine balancing act: maintaining tolerance to the fetal allograft while restoring innate and adaptive immune mechanisms for protection against microbial challenges. Successful implantation, placental development, and fetal growth, as well as the maintenance of maternal and fetal health, hinge on the delicate balance of the maternal immune response. An excessive activation of the immune system amplifies the risk of fetal rejection and adverse pregnancy outcomes such as preterm labor and birth, recurrent pregnancy loss, fetal growth restriction, gestational diabetes mellitus and gestational hypertension including preeclampsia and HELLP (Hemolysis, Elevated Liver enzymes and Low Platelets) syndrome. Immune system disturbances can precipitate maternal or fetal infection. Crosstalk between innate and adaptive immunity requires appropriate regulation of both immune wings during pregnancy. Studies elucidating the complexity of molecular and cellular interactions at the maternal-fetal interface and how these interactions are modulated favorably are crucial in developing targeted therapeutics. This Research Topic aims to shed light on these unclear mechanisms that are crucial for the development of novel diagnostic tools and innovative therapeutic approaches. In this series, original research papers, case reports and comprehensive reviews elaborate the importance of innate immune cells, complement system and link between innate and adaptive immunity in facilitating a normal pregnancy; failure to do so contribute to the pathophysiology of adverse pregnancy.

The maternal-fetal interface is still considered an enigmatic district from the immunological point of view; the complex biological mechanisms are yet to be fully elucidated. Decidua is likely to be the primary site of replication for several TORCH [toxoplasmosis, others (Syphilis, Hepatitis B), rubella, Cytomegalovirus (CMV), and herpes simplex] pathogens and could form a reservoir for pathogens. Thus, to create an immune tolerant environment towards the nonself-embryonic antigens while acting simultaneously as a barrier to defend the fetus from the attack by pathogens is a crucial job.

A review article by Zang et al. highlights the inflammatory response with disproportionate number of immune cells and cytokine imbalance in the maternal-fetal interface in chronic histiocytic intervillositis (CHI). It indicates dysregulated expression of HLA in the extravillous trophoblasts: upregulation of HLA-A/B and downregulation of HLA-G. A concomitant decline in regulatory T cell (Treg) number leads to the breakdown of immune tolerance at the maternal-fetal interface in CHI. In this review, a two-stage model is proposed where the first phase involves downregulation of CD200, CD30 and ICAM expression on syncytiotrophoblasts that initiates an inflammatory response. This leads to the infiltration of maternal CD68^+^ macrophages, releasing high levels of IL-1β and IL-6 in the intervillous space and activation of CD4^+^T and CD8^+^T cells. In the second phase of the tissue repair, M1 macrophages polarize to M2 phenotype synthesizing TGF-β. Chronic inflammation leads to placental tissue damage, activating complement. C4d deposition is observed in the intercellular spaces in CHI that can further promote fibrinogen to fibrin conversion. Massive fibrin deposition around the chorionic villi could become a hindrance to maternal-fetal blood supply leading to pathological conditions such as intra-uterine fetal growth restrictions (IUGR) and recurrent pregnancy loss (RPL). Maternal circulating exosomes, microRNAs, HLA antibodies and cell free fetal DNA are the future diagnostic biomarkers for CHI (1).

In the list of biomarkers for Recurrent Pregnancy Loss (RPL) the work by Wu et al. includes LYN (Src family tyrosine kinase), a crucial immune cell signaling regulator expressed on hematopoietic cells and CYBB (Cytochrome B-245 Beta Chain) that encodes protein critical for ROS generation. Transcriptome analysis indicates downregulated expression of LYN and CYBB in the endometrium of subjects compared to normal endometrium. LYN expression exhibited a positive correlation with M2 macrophages and CYBB with CD8^+^ T cells, whereas negative correlation was observed with eosinophils for LYN and naïve B cells for CYBB. LYN and CYBB genes can influence the inflammatory response and dysregulated-signaling mechanisms can drive RPL and thus, can be potential biomarkers for the diagnosis of RPL (2).

Despite recent advancement, some adverse conditions during pregnancy such as Feto-maternal hemorrhage (FMH), remain underdiagnosed. FMH is a pathological condition where fetal blood meets the maternal blood due to the rupture of placenta causing anemia, neurological injury and even fetal death. Hao et al. reported a case of severe anemia in term neonate delivered at 38 weeks of gestation with Rh incompatibility, and 2% of fetal erythrocytes in maternal blood. However, depending on the preliminary evaluation, this case was previously excluded from infectious and nutritional etiologies. This emphasizes the importance of simple blood group examination integrated with flow cytometry in the detection of FMH cases (3).

Complement system plays an indispensable role in pregnancy and parturition. Complement proteins can be found in the fallopian tubes, cervices, uterus and placenta, in order to eliminate pathogens. During pregnancy, complement activity is physiologically upregulated, mostly to perform clearance of apoptotic trophoblasts during placental remodeling. A fine balance between complement activation maintained by complement regulatory proteins across the placenta and in the serum affects the fate of a healthy pregnancy as reported by Roy et al (4). Two soluble complement regulatory proteins, properdin that can upregulate complement alternative pathway, and factor H (FH) that can downregulate complement activation, were shown to have contrasting levels in the placenta during preeclampsia (PE) and RPL. Properdin, along with C3 and C5, was significantly higher, while FH significantly lower in the placenta and umbilical cord of PE mothers compared to healthy placentae, so is the case in RPL. However, in gestational diabetes mellitus, properdin as well as FH were at significantly higher levels, but not C3 and C5. Thus, the expression of the two complement regulators can be differential, controlling the pathological dynamics depending on the etiology of the disease. Another study by Yasmin et al., explored further the role of properdin in PE. Though properdin is maintained at a high level in the PE placentae, its levels in the serum and in the placental circulatory exosomes of PE mothers were significantly lower compared to healthy counterparts. Syncytial knots were loaded with properdin in the PE placentae with a high proportion of pyknotic nuclei and cleaved caspase-3. This association of properdin with apoptotic nuclei in PE placenta strengthens the role of properdin as an inflammatory marker in PE (5).

Successful implantation and pregnancy depend on finely tuned immune adaptations at the endometrial and the systemic level. The pregnant uterus is characterized by a high number of Natural Killer (NK) cells and macrophages with a low number of dendritic cells and Tregs. The interaction between immune cells, decidual stromal cells, and trophoblasts constitute a vast network of cellular connections at the maternal-fetal interface. A few articles in the Research Topic highlight how dysregulation of immune cell populations, particularly NK cells and T cells, can compromise reproductive success, while also pointing to potential diagnostic, therapeutic, and prognostic avenues.

A study on endometrial immune assessment in patients with previous euploid blastocyst failure underscores a critical role of local immune balance during the mid-luteal phase. A retrospective analysis compared outcomes between patients having their first euploid transfer, those with previous euploid failure, and a study group receiving modified endometrial preparation after immune profiling. The results showed significant differences between first attempts and previous failures without testing. Despite the transfer of chromosomally normal embryos, implantation and live birth rates were significantly reduced in women with prior euploid failures, suggesting that the embryo quality alone is not sufficient to ensure a successful pregnancy. Importantly, patients who underwent endometrial immune profiling and received a personalized preparation regimen targeting uterine NK (uNK) cell recruitment, maturation, and activity showed outcomes comparable to first-attempt patients. This study highlights endometrial immune dysfunction as a modifiable contributor to implantation failure and supports personalized immune-based interventions (6). 

The importance of NK cell regulation is echoed in another study examining moderate-intensity aerobic exercise in women with unexplained RPL. Here, a three-month exercise intervention induced significant phenotypic and functional changes in peripheral NK cells, including reduced expression of activating and inhibitory receptors (CD161, NKp30 and NKG2A) and lower IFN-γ production, indicative of a less pro-inflammatory profile. Where uNK cells were not significantly altered, the results suggest that systemic immune modulation through lifestyle interventions may indirectly support reproductive tolerance, complementing more targeted endometrial approaches (7).

Immune perturbations during pregnancy are further illustrated by a single-cell RNA-sequencing study of post-COVID-19 pregnant women, which demonstrated sustained impairment of NK cell numbers and cytotoxic function even after recovery from the infection. Attenuated interferon signaling and reduced expression of perforin and granzyme B suggest long-lasting immune remodeling that can influence placental defense, inflammation, and possibly pregnancy outcomes. These findings emphasize how external insults, such as viral infections, can disrupt the delicate immune equilibrium required for healthy gestation (8).

Murine models often fail to mimic the true microenvironment of the diverse subpopulation of T cells at the maternal-fetal interface. Whillock et al. cohoused female pet store mice with female B6 mice for more than four weeks. Then these female B6 mice, which had natural microbial exposure, were mated with male B6 mice (natural microbial exposure mouse model, NME). By mimicking human microbial environments, this model revealed increased diversity and abundance of effector, memory, tissue-resident, and unconventional T cell populations at the maternal-fetal interface. These adaptations mirror human decidua and highlight the role of physiological immune stimulation in promoting tolerance while maintaining antimicrobial defense. The number and the diversity of both effector and memory CD4^+^T and CD8^+^T increased in NME models, closely resembling the human placental population of CD8^+^T cells (9).

Finally, the study on pre-partum blood leukocyte profiles extends immune assessment into late pregnancy, demonstrating that non-reductionist analysis of leukocyte interactions can define inflammatory stages predictive of adverse birth outcomes such as preterm birth and low birth weight. This retrospective study considered blood samples from 131 pregnant women in their 2^nd^ and 3^rd^ trimester stages. The study characterized four inflammatory stages based on phagocyte/lymphocyte ratio where the double risk observations were associated with high serum ferritin. This approach reinforces the idea that immune balance across pregnancy stages, and not isolated markers, determines outcomes and offers a low-cost, translational prognostic tool (10).

Together, these studies converge on a central theme: the success of implantation and pregnancy is tightly linked to a dynamic, context-dependent immune regulation. Advances in immune profiling, personalized interventions, lifestyle modulation, and systems-level analysis collectively offer promising avenues to improve fertility treatments and pregnancy outcomes.
